# Therapeutic potential of dihydroartemisinin in mitigating radiation‐induced lung injury: Inhibition of ferroptosis through Nrf2/HO‐1 pathways in mice

**DOI:** 10.1002/iid3.1175

**Published:** 2024-02-05

**Authors:** Xin Ning, Weidong Zhao, Qiaoyuan Wu, Cailan Wang, Shixiong Liang

**Affiliations:** ^1^ Department of Radiation Oncology Guangxi Medical University Cancer Hospital Nanning Guangxi Zhuang Autonomous Region China

**Keywords:** dihydroartemisinin, ferroptosis, Nrf2/HO‐1 pathways, radiation‐induced lung injury

## Abstract

**Background:**

Radiation‐induced lung injury (RILI) is a common consequence of thoracic radiation therapy that lacks effective preventative and treatment strategies. Dihydroartemisinin (DHA), a derivative of artemisinin, affects oxidative stress, immunomodulation, and inflammation. It is uncertain whether DHA reduces RILI. In this work, we investigated the specific mechanisms of action of DHA in RILI.

**Methods:**

Twenty‐four C57BL/6J mice were randomly divided into four groups of six mice each: Control group, irradiation (IR) group, IR + DHA group, and IR + DHA + Brusatol group. The IR group received no interventions along with radiation treatment. Mice were killed 30 days after the irradiation. Morphologic and pathologic changes in lung tissue were observed with hematoxylin and eosin staining. Detection of hydroxyproline levels for assessing the extent of pulmonary fibrosis. Tumor necrosis factor α (TNF‐α), transforming growth factor‐β (TGF‐β), glutathione peroxidase (GPX4), Nuclear factor erythroid 2‐related factor 2 (Nrf2), and heme oxygenase‐1 (HO‐1) expression in lung tissues were detected. In addition, mitochondrial ultrastructural changes in lung tissues were also observed, and the glutathione (GSH) content in lung tissues was assessed.

**Results:**

DHA attenuated radiation‐induced pathological lung injury and hydroxyproline levels. Additionally, it decreased TNF‐α and TGF‐β after irradiation. DHA may additionally stimulate the Nrf2/HO‐1 pathway. DHA upregulated GPX4 and GSH levels and inhibited cellular ferroptosis. Brusatol reversed the inhibitory effect of DHA on ferroptosis and its protective effect on RILI.

**Conclusion:**

DHA modulated the Nrf2/HO‐1 pathway to prevent cellular ferroptosis, which reduced RILI. Therefore, DHA could be a potential drug for the treatment of RILI.

## Graphical abstract

In this study, we found that post‐radiation mice showed significant lung inflammation and ferroptosis. Through the Nrf2/HO‐1 pathway, dihydroartemisinin (DHA) prevented ferroptosis and reduced radiation‐induced lung injury (RILI). According to our research, ferroptosis and the Nrf2/HO‐1 pathway could be potential targets for the treatment of RILI, and DHA may be an applicable drug in RILI prevention and treatment

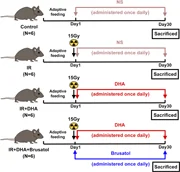

## 1 INTRODUCTION

Radiotherapy has played a crucial role in oncology for several years. Radiation therapy for thoracic cancers frequently results in radiation‐induced lung injury (RILI). There are two stages of RILI: radiation pneumonia and radiation pulmonary fibrosis. The characteristic pathophysiological changes include epithelial and endothelial cell injury, inflammatory cell infiltration, cytokine release, fibroblast differentiation, extracellular matrix deposition, and collagen synthesis.^1^, ^2^, ^3^ This chain of events results in imaging abnormalities and the development of clinical symptoms. Ultimately, it may lead to respiratory distress and significantly reduce the patient's quality of life. However, there is a lack of any efficient clinical treatments or preventions for RILI now. In recent years, some studies have found that ferroptosis is associated with the development of RILI.^4^ Perhaps targeting ferroptosis could be an effective therapeutic direction in the future.

A unique type of cell death, the ferroptosis, has been increasingly studied in recent years. Compared to apoptosis and autophagy, cytological changes are the primary characteristics of ferroptosis. Ferroptosis is induced by an increase in lipid reactive oxygen groups and a decrease in glutathione peroxidase 4 (GPX4) activity.^5^ The onset of membrane lipid peroxidation and oxidative stress leads to a selective decrease in plasma membrane permeability, finally causing cytological alterations.^6^ GSH is an intracellular antioxidant that inhibits reactive oxygen species and combats inflammatory damage.^7^ The nuclear factor erythroid 2‐related factor 2 (Nrf2) is a key regulator of cellular antioxidant responses and plays a critical role in attenuating lipid peroxidation and ferroptosis.^8^ The antioxidant gene heme oxygenase‐1 (HO‐1), one of the downstream target genes of Nrf2, is also involved in the antioxidative stress process in organisms.^9^ Several studies have shown in recent years that Nrf2/HO‐1 is associated with ferroptosis.^10^, ^11^, ^12^ Studies have found that ferroptosis was also closely related to RILI.^4^, ^13^ Therefore, it is crucial to investigate the role of Nrf2/HO‐1 in ferroptosis and develop new drugs for the treatment of RILI.

From the Chinese herb *Artemisia annua*, the active component artemisinin is extracted. The primary metabolite of artemisinin and its derivatives is dihydroartemisinin (DHA). DHA is more potent and stable and has a lower recurrence rate during treatment than that of artemisinin.^14^, ^15^ DHA has antimalarial properties, but it also affects inflammation,^16^ immune function,^17^ angiogenesis,^18^ and oxidative stress.^19^ A study found that DHA attenuated acute lung injury in mice in an Nrf2‐dependent manner.^20^ Previous research demonstrated that DHA protects against pulmonary inflammation and oxidative stress in mice with early RILI.^21^ DHA has been shown to reduce lung injury and pulmonary fibrosis by reducing alveolar inflammation.^22^ These indicated that DHA has some protective effects in lung injury. However, the specific protective effect of DHA on RILI remains not well understood.

In this study, we constructed a mouse model of RILI to investigate the protective effects of DHA against RILI, to explore whether ferroptosis was involved in this pathologic process, and its potential mechanisms in vivo.

## 2 MATERIALS AND METHODS

### 2.1 Materials

DHA and Brusatol (Nrf2 inhibitor) were purchased from Tauto. GSH assay kits and antibodies against β‐actin were purchased from Beyotime. The hydroxyproline kit was purchased from the Jiancheng Bioengineering Institute. The TNF‐α and TGF‐β ELISA kits were purchased from Elabscience. Antibodies against HO‐1 were purchased from Abcam. Antibodies against tumor necrosis factor α (TNF‐α), transforming growth factor‐β (TGF‐β), GPX4, Nrf2, electron microscope fixative, hydrogen peroxide (H_2_O_2_), hematoxylin, eosin, osmium acid, lead citrate, uranium acetate and the immunohistochemistry (IHC) special goat anti‐rabbit secondary antibody were purchased from Servicebio. The goat anti‐rabbit IgG (H + L) fluorescent secondary antibody was purchased from Cell Signaling Technology; excitation is 777 nm and peak fluorescence emission is 794 nm. The diaminobenzidine (DAB), sodium dodecyl sulfate (SDS), radioimmunoprecipitation assay (RIPA) buffer and Tris Buffered Saline Tween (TBST) were purchased from Solarbio. The polyvinylidene fluoride membrane was purchased from Millipore. The acetone and ethanol were purchased from Chron Chemicals.

### 2.2 Animals and model

Adult male C57BL/6 mice (25–30 g, 6–8 weeks) were purchased from Guangxi Medical University Animal Experiment Center. The mice were housed in a ventilated environment with ad libitum feeding and watering at a constant temperature of 24 ± 1°C. Experiments were conducted after 1 week of adaptive rearing.

**Figure 1:**
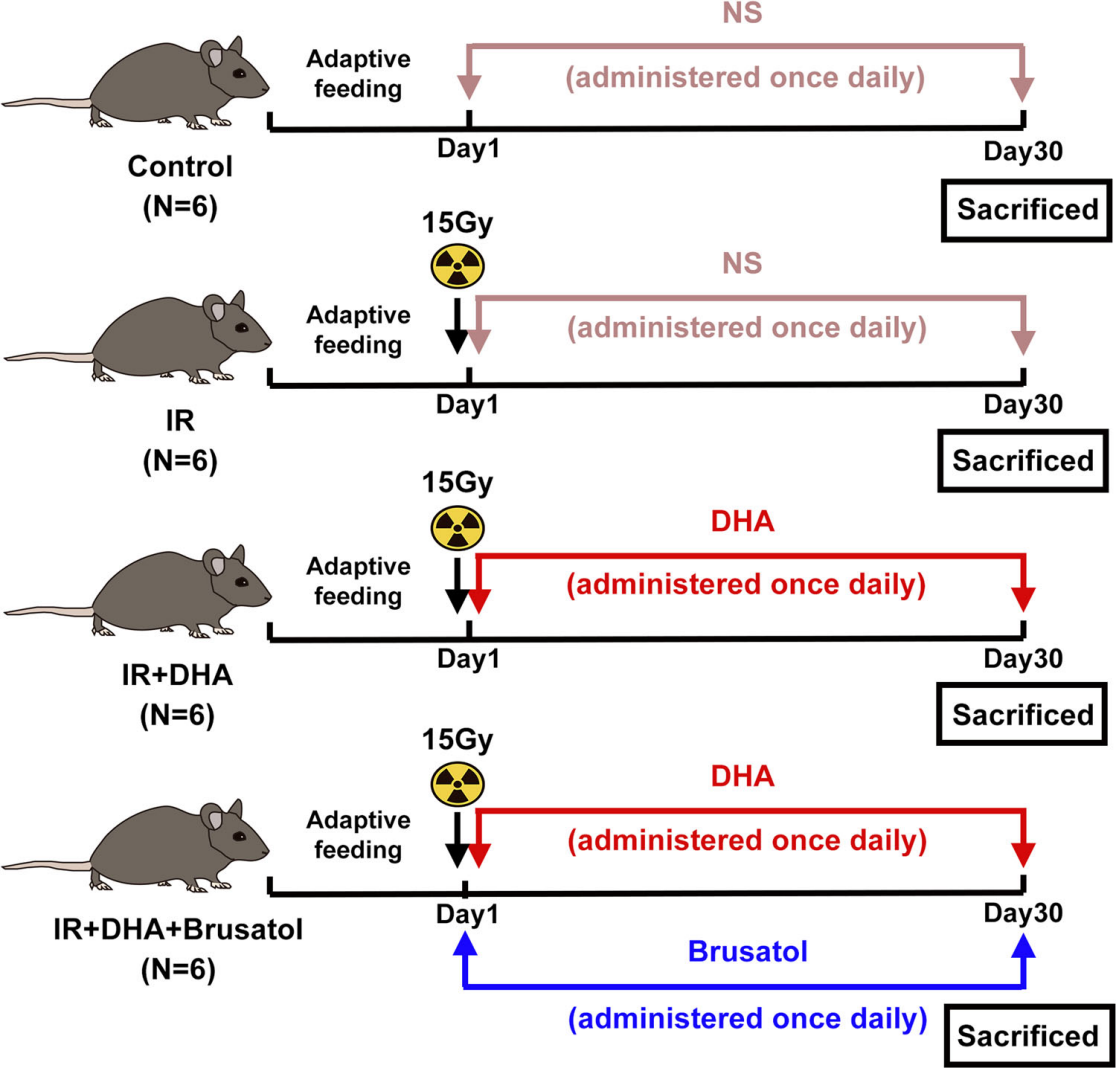
Experimental design. DHA, dihydroartemisinin; IR, irradiation; IR + DHA, irradiation + DHA; IR + DHA + Brusatol, irradiation + DHA + Brusatol; NS, normal saline.

Twenty‐four C57BL/6 mice were divided into four groups at random (*n* = 6), Control group, irradiation (IR) group (irradiation alone), IR + DHA group (irradiation and administered DHA), and IR + DHA + Brusatol group (irradiation, administered DHA and Brusatol). The other three mouse groups, aside from the Control group, were modeled for RILI (the modeling procedure was performed according to prior studies^21^): The mice were anesthetized by intraperitoneal injection of 1% sodium pentobarbital (50 mg/kg). After anesthesia, the mice were placed under a linear gas pedal (Versa HD, Elekta) and then irradiated with a single vertical thorax irradiation with 6MV‐X rays at a dose of 15 Gy. The irradiation field was 2.0 × 2.0 cm^2^, and the source of radiation was 6MV‐X rays. Saline was administered to the Control and IR groups following irradiation. The IR + DHA group received daily intragastric injection of DHA (50 mg/kg), and the IR + DHA + Brusatol group received daily intragastric injection of DHA (50 mg/kg) and intraperitoneal injection of Brusatol (2 mg/kg). The treatment was continued for 30 consecutive days. The experimental design is shown in Figure 1.

### 2.3 Sample collection

Tissue sampling was performed on the 30th day following irradiation. After anesthetizing the mice, the thoracic cavity was exposed, and heart blood was extracted. The blood was left to clot for 2 h at room temperature. After allowing the serum to spontaneously precipitate, it was centrifuged for 10 min (4°C, 665 RCF). The obtained serum was stored in a freezer at −80°C. Fresh lung tissue was removed bilaterally, and the right lung tissue was rapidly dissected. A piece of tissue measuring 1 mm^3^ was removed from the right lung, treated with 2.5% glutaraldehyde, and investigated by transmission electron microscope (TEM). The prepared sections were examined for histological changes and for immunohistochemical analysis. The remaining lung tissue was kept in freezing tubes and stored in a freezer at −80°C and used for subsequent assays.

### 2.4 Hematoxylin and eosin staining

From treated mice, lung tissue samples were taken, fixed with 4% paraformaldehyde, and then cut into sections using paraffin embedding. Light microscopy was used to see the pathological alterations after the slices underwent hematoxylin and eosin (H&E) staining. Lung injury was assessed using a semi‐quantitative scoring system, according to the American Thoracic Society symposium report.^23^ Pathologists without knowledge of the experimental groups assessed a randomly selected high‐magnification field (400× magnification). For each sample, a total of five nonoverlapping places were chosen at random, and the mean of the total score of these five fields was considered as the final pathological score.

### 2.5 Hydroxyproline assay

Hydroxyproline kit was used to determine hydroxyproline levels. The steps on the kit instructions were followed step‐by‐step. Finally, the optical density (OD) was measured at 560 nm. The formula for calculating hydroxyproline level is: hydroxyproline content (μg/mg wet weight) = [(measured OD value − blank OD value)/(standard OD value − blank OD value)] × standard content (5 μg/mL) × (total volume of hydrolysate 10 ml/mg tissue wet weight).

### 2.6 IHC analysis

Dewaxed in xylene, the paraffin slices were dehydrated in a gradient ethanol solution. To limit endogenous peroxidase activity, the slices were treated with 3% H_2_O_2_, then blocked with goat serum. Antibodies against TNF‐α, TGF‐β, GPX4, and Nrf2 were then applied to slices and kept at 4°C overnight. The color presentation was seen by adding the DAB color developer dropwise after 1 h of secondary antibody incubation. Hematoxylin was used as a counterstain, and sections were dried out and sealed. Each segment was photographed under a light microscope. The optical density was evaluated using Image Pro Plus (Media Cybernetics). The immunostaining data were represented as average optical density (AOD), and AOD = integrated optical density (IOD)/area (%).

### 2.7 Elisa assay

TGF‐β and TNF‐α levels in mice alveolar lavage fluid were measured using the Mouse Elisa Kit. All steps were performed according to the instructions and OD values were measured at 450 nm using an enzyme meter (Thermo).

### 2.8 Western blot analysis assay

Lung tissue was collected and lysed using RIPA lysis solution. Total tissue protein was extracted by centrifuging tissue homogenates for 10 min (4°C, 15,500 RCF) after they had been prepared on ice. The samples were electrophoresed, and the membrane was transferred in an SDS‐polyacrylamide gel electrophoresis bath. The protein was transferred using a polyvinylidene fluoride membrane. The membrane was confined at room temperature for 30 min. Followed by overnight incubation with rabbit‐derived primary antibody β‐actin (1:1000) and HO‐1 (1:1000) at 4°C. TBST was used to thoroughly clean the membrane three times. The membrane was then incubated with goat anti‐rabbit IgG (H + L) fluorescent secondary antibody (1:20,000) at room temperature for 1 h. Protein strips were scanned using the ODYSSEY CLx (dual‐color infrared laser imaging system) scanner. To analyze and assess the bands, ImageJ software (National Institutes of Health) was utilized.

### 2.9 Transmission electron microscope

Pieces of lung tissue measuring 1 mm^3^ were collected and fixed for 2 h at 4°C in an electron microscope fixative. They were then fixed for 2 h in 1% osmium acid. Following that, sections were dehydrated in acetone and put in an ethanol gradient. The sections were implanted, divided into sections, stained with lead citrate and 2% uranium acetate, and allowed to dry at room temperature for an entire night. Finally, photographs of the sample taken using a TEM (Hitachi HT7700) and evaluated (Hitachi TEM system) were assembled.

### 2.10 Measurement of GSH level

After the preparation of mouse serum, various reagents were added sequentially according to the kit instructions. An enzyme marker was used to quantify the absorbance at 412 nm after mixing and incubation at room temperature for 2 min. The absorbance value of the blank tube is A1, and the absorbance value of the measurement tube is A2. The GSH content in the mouse serum was then calculated using the following formula: GSH (μ mol/mL) = 1.334 × (A2 − A1).

### 2.11 Statistical analysis

The GraphPad Prism 8 program (GraphPad Prism Software) was used for all statistical analyses. Statistical results were expressed as mean ± standard deviation (SD). The Shapiro–Wilk test was used to confirm the normal distribution of the data. One‐way ANOVA and a Turkey multiple comparisons posttest was used for comparison between groups. Differences were considered statistically significant at *p* < .05.

## 3 RESULTS

### 3.1 DHA relieves lung injury in RILI

**Figure 2:**
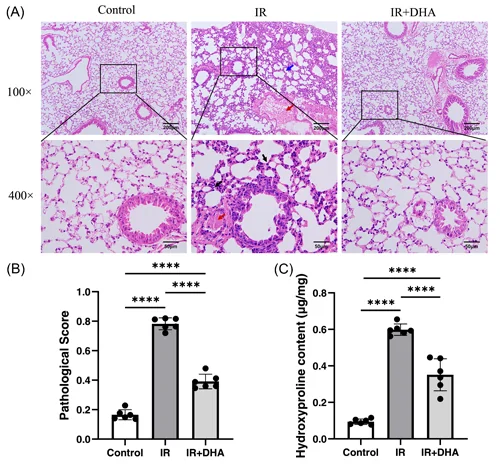
Effect of DHA on histopathological changes in mice lung tissues. (A) Images of representative H&E‐stained lung sections of mice. (B) Pathological score statistics of the three experimental groups. (C) Hydroxyproline content of lung tissue in mice. DHA, dihydroartemisinin; H&E, hematoxylin and eosin; IR, irradiation; IR + DHA, irradiation + DHA. Blue arrow, thickened alveolar interstitium; Red arrow, inflammatory exudate; Black arrow, inflammatory cell infiltration. Original magnification 100×, 400×. Data are presented as mean ± standard deviation, *p < .05, **p < .01, ***p < .001, ****p < .0001. Differences in statistical significance between groups were determined by one‐way ANOVA and a Turkey multiple comparisons posttest.

We established a mouse model of RILI using a single whole‐lung irradiation at 15 Gy. Lung injury was evaluated according to the pathological score reported by the American Thoracic Society Symposium.^23^ Our results demonstrated that the control group mice had a clear lung texture, normal alveolar structure, and no obvious bleeding or rupture of the alveolar wall. Mice in the IR group had exudates in the alveoli, disorganized alveolar structures, and significantly thickened alveolar septa than the control group. The alveolar interstitial and cavity were found to contain a substantial number of inflammatory cells. Comparing the IR + DHA group to the IR group, lung tissue damage, and inflammatory cell infiltration were decreased (Figure 2A). The pathologic scores in the IR group were significantly higher than those in the Control group (*p* < .0001), whereas the pathologic scores in the IR + DHA group were lower than those in the IR group (*p* < .0001) but lower than those in the Control group (*p* < .0001) (Figure 2B). The results of hydroxyproline content measurement showed that the hydroxyproline content was significantly higher in the IR group compared to the Control group (*p* < .0001). The hydroxyproline content in the IR + DHA group decreased significantly (*p* < .0001) compared to the IR group but was still higher than that in the Control group (Figure 2C). It can be concluded that DHA can alleviate some of the radiation‐induced lung inflammation and lung fibrosis.

**Figure 3:**
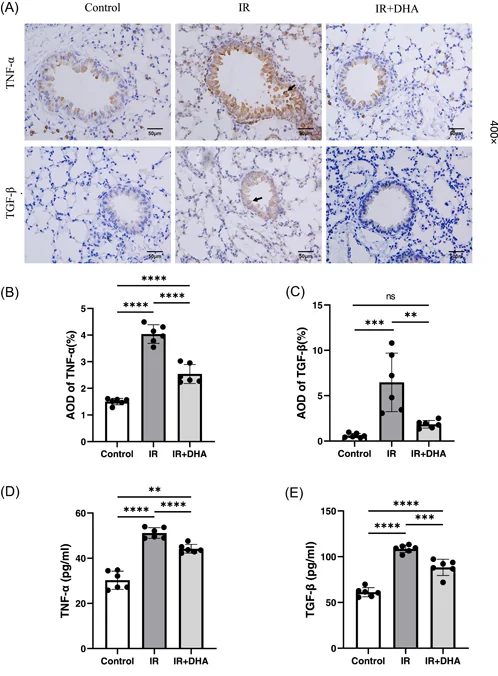
Effect of DHA on inflammatory cytokines, TNF‐α and TGF‐β, in lung tissues of various groups of mice. (A) Representative images of IHC staining for TNF‐α and TGF‐β in mice lung tissue; (B) AOD value of TNF‐α expressions; (C) AOD value of TGF‐β expressions; (D) TNF‐α in BALF was detected by ELISA; (E) TGF‐β in BALF was detected by ELISA. AOD, average optical density; BALF, bronchoalveolar lavage fluid; DHA, dihydroartemisinin; ELISA, enzyme‐linked immunosorbent assay; IHC, immunohistochemistry; IOD, Integrated optical density; TGF‐β, transforming growth factor‐β; TNF‐α, tumor necrosis factor α. AOD = IOD/Area (%). IR, irradiation; IR + DHA, irradiation +DHA. Black arrow, positive region; Red arrow, weak positive area. Original magnification 400×. Data are presented as mean ± standard deviation; *p < .05, **p < .01, ***p < .001. Differences in statistical significance between groups were determined by one‐way ANOVA and a Turkey multiple comparisons posttest.

We detected the expression levels of TNF‐α and TGF‐β through IHC assay and ELISA assay. The IHC assay results demonstrated that TNF‐α and TGF‐β were mainly expressed in the bronchial epithelial cells, with a lower expression observed in the lung parenchyma (Figure 3A). TNF‐α and TGF‐β levels were elevated in the IR group compared to the Control group (*p* < .0001, *p* = .0002). TNF‐α and TGF‐β levels were decreased in the IR + DHA group compared to the IR group (*p* < .0001, *p* = .0019) (Figure 3B,C). The results of Elisa assay also showed that the levels of TNF‐α and TGF‐β were higher in the IR group compared to the Control group (*p* < .0001, *p* < .0001), and the levels of TNF‐α and TGF‐β were lower in the IR + DHA group compared to the IR group (*p* < .0001, *p* = .0002) (Figure 3D,E), which was in agreement with the results of IHC assay, suggesting that DHA therapy attenuated levels of TNF‐α and TGF‐β and reduced lung injury.

### 3.2 DHA upregulates the Nrf2/HO‐1 pathway

**Figure 4:**
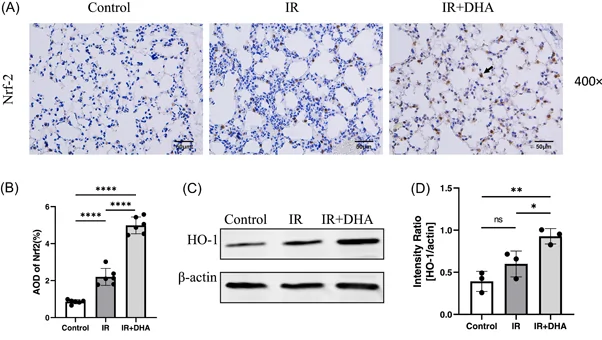
Effects of DHA on Nrf2 and HO‐1 expressions in lung tissues of various groups of mice. (A) Representative images of IHC staining for Nrf2 in mice lung tissues; (B) AOD of Nrf2 expressions; (C) Western blots for HO‐1 in mice lung tissues; (D) The relative expression of HO‐1 determined by optical densitometry. AOD, average optical density; DHA, dihydroartemisinin; HO‐1, Heme oxygenase‐1; IHC, immunohistochemistry; IOD, Integrated optical density; IR, irradiation; Nrf2, nuclear factor erythroid 2‐related factor 2; IR + DHA, irradiation + DHA. AOD = IOD/Area (%). Black arrow, positive cell. Original magnification 400×. Data are presented as mean ± standard deviation, *p < .05, **p < .01, ***p < .001, ****p < .0001. Differences in statistical significance between groups were determined by one‐way ANOVA and a Turkey multiple comparisons posttest.

We investigated the effect of DHA on Nrf2/HO‐1 by detecting Nrf2 and HO‐1 expression in mice lung tissue. According to the experimental findings, the expression levels of Nrf2 were increased in the IR group compared with the control group (Figure 4A,B) (*p* < .0001), while there was no significant difference in the expression level of HO‐1 expression (Figure 4C,D) (*p* = .1844). DHA treatment significantly increased the expression of Nrf2 and HO‐1 when compared to the IR group (*p* < .0001 and *p* = .0411) (Figure 4B,D), demonstrating that DHA activated the expression of Nrf2 and HO‐1.

### 3.3 DHA suppresses ferroptosis by mediating Nrf2/HO‐1 pathway

**Figure 5:**
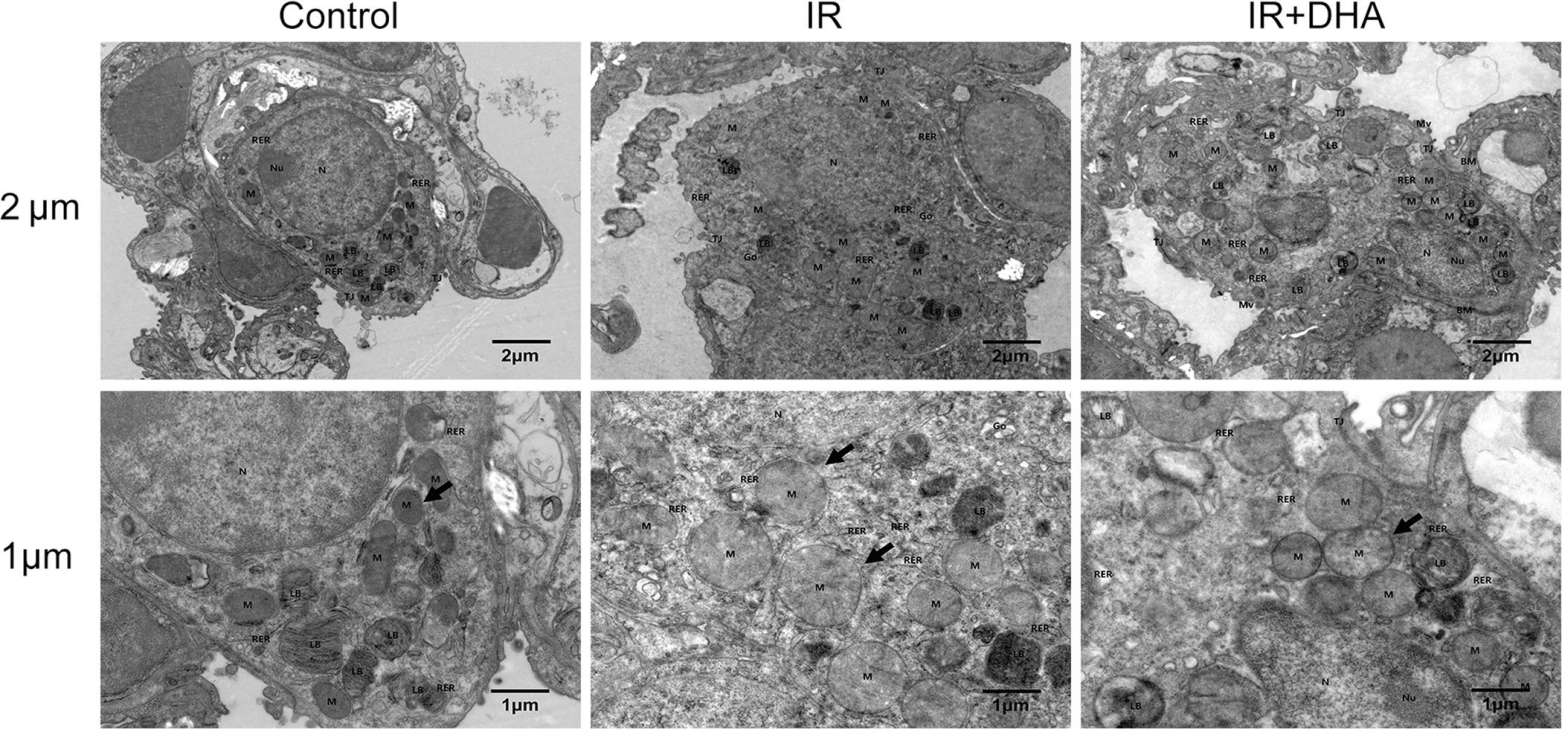
Effect of DHA on the ultrastructure of mice lung tissue cells. The ultrastructure of mouse lung tissue cells was examined using TEM on Day 30. DHA, dihydroartemisinin; IR, irradiation; TEM, transmission electron microscope. IR + DHA, irradiation + DHA; Black arrow, Mitochondria. Upper panel, scale bar, 2 μm; lower panel, scale bar, 1 μm.

The ultrastructure of the mice's lung cells was observed using TEM on day 30. In the control group, the experimental findings showed no obvious harm to alveolar type II epithelial cells. The cell membranes remained intact. The mitochondrial structures of the cells were normal. Mice in the IR group had significantly edematous alveolar type II epithelial cells with membrane lysis and a markedly damaged ultrastructure. Mitochondria were swollen. The mitochondrial membrane was broken and disintegrated, the matrix was spilled out, and cristae were broken and reduced. The IR + DHA group showed less cellular ultrastructural damage and a decline in alveolar type II epithelial cells than the IR group (Figure 5). These after‐irradiation characteristics of mitochondrial ferroptosis provided evidence that ferroptosis had been involved in the emergence of RILI. The experimental results showed that treatment with DHA inhibited ferroptosis in mice lung tissue cells.

**Figure 6:**
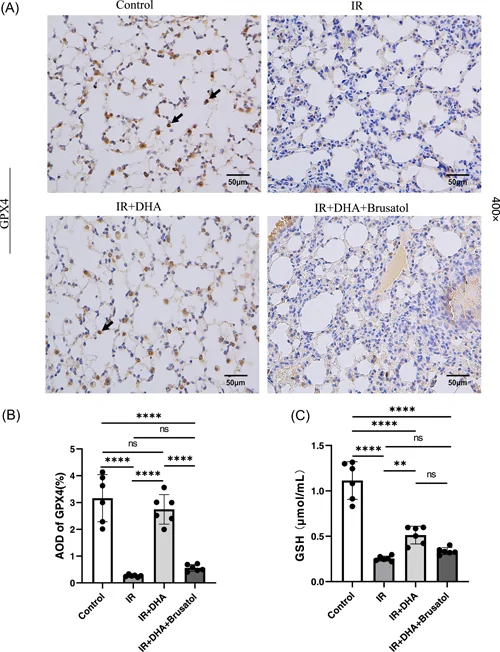
Effect of DHA and Brusatol on the levels of biomarkers of ferroptosis in mice lung tissues. (A) Representative images of IHC staining for GPX4 in mice lung tissues; (B) AOD of GPX4 expressions; (C) The GSH levels in mice serum. AOD, average optical density; DHA, dihydroartemisinin; GPX4, glutathione peroxidase 4; GSH, glutathione; IHC, immunohistochemistry; IOD, integrated optical density; IR, irradiation. IR + DHA, irradiation + DHA; IR + DHA + Brusatol, irradiation + DHA + Brusatol. AOD = IOD/Area (%). Black arrow, positive cell. Original magnification 400×. Data are presented as mean ± standard deviation, *p < .05, **p < .01, ***p < 0.001, ****p < .0001. Differences in statistical significance between groups were determined by one‐way ANOVA and a Turkey multiple comparisons posttest.

From the above experiments, we demonstrated that DHA could inhibit ferroptosis. Additionally, we demonstrated the role of Nrf2 in ferroptosis using Brusatol, an Nrf2 inhibitor. We assayed for ferroptosis by measuring the levels of GPX4 and GSH. Experimental results showed that the IR group showed significantly lower GPX4 expression (Figure 6A,B) and GSH levels (Figure 6C) compared to the control group (*p* < .0001, *p* < .0001). In contrast, GPX4 expression and GSH levels were significantly upregulated in the lung tissues of IR + DHA mice compared with the IR group (*p* < .0001, *p* = .0052). In the IR + DHA + Brusatol group as compared to the IR + DHA group, GPX4 protein expression was considerably downregulated (*p* < .0001), and GSH levels were decreased, but there was no significant difference (*p* = .0720) (Figure 6B,C). The experimental results showed that DHA treatment inhibited ferroptosis in mouse lung tissue cells, while inhibition of the Nrf2 pathway reduced the inhibitory effect of DHA on cellular ferroptosis.

### 3.4 DHA suppresses lung inflammation by mediating Nrf2/HO‐1 pathway

**Figure 7:**
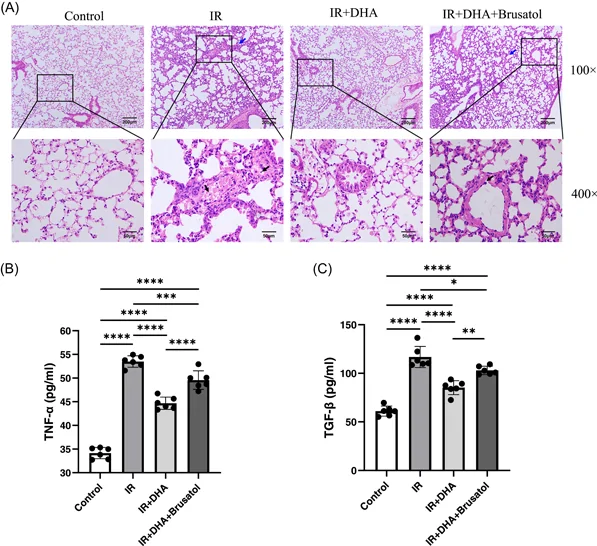
Effects of DHA and Brusatol on radiation‐induced lung inflammation in mice. (A) Images of representative H&E‐stained lung sections of mice; (B) TNF‐α in BALF was detected by ELISA; (C) TGF‐β in BALF was detected by ELISA. BALF, bronchoalveolar lavage fluid; DHA, dihydroartemisinin; ELISA, enzyme‐linked immunosorbent assay; H&E, hematoxylin and eosin; IR, irradiation; TGF‐β, transforming growth factor‐β; TNF‐α, tumor necrosis factor α. IR + DHA, irradiation +DHA; IR + DHA + Brusatol, irradiation + DHA + Brusatol. Blue arrow, thickened alveolar interstitium; Black arrow, inflammatory cell infiltration. Original magnification 100×, 400×. Data are presented as mean ± standard deviation, *p < .05, **p < .01, ***p < .001, ****p < .0001. Differences in statistical significance between groups were determined by one‐way ANOVA and a Turkey multiple comparisons posttest.

The results of the experiment showed that the lung tissue structure was normal in the blank group, and the alveolar septum was thickened and infiltrated with inflammatory cells in the IR group. the alveolar septum thickening was reduced in the IR + DHA group compared to the IR group, but there were still some inflammatory cells infiltrated. the alveolar septum was thickened and infiltrated with inflammatory cells in the IR + DHA + Brusatol group compared to the IR + DHA group (Figure 7A). In addition, we examined the levels of TNF‐α and TGF‐β factors in alveolar lavage fluid. The results showed that the levels of both factors were significantly higher in the IR and IR + DHA + Brusatol groups compared to the control group (*p* < .0001, *p* < .0001). And the levels of both factors were significantly lower in the IR + DHA group compared to the IR group (*p* < .0001, *p* < .0001), but still higher than the control group (*p* < .0001, *p* < .0001). In contrast, the levels of both factors were again elevated in the IR + DHA + Brusatol group compared to the IR + DHA group (*p* < .0001, *p* = .0023) (Figure 7B,C). These results suggest that DHA can effectively alleviate some of the RILI and attenuate the levels of TNF‐α and TGF‐β, whereas Brusatol will this effect of DHA.

## 4 DISCUSSION

During radiation therapy for chest tumors, the lung tissue, a moderately radiation‐sensitive organ, often suffers from RILI.^24^ RILI usually occurs weeks to months after irradiation.^2^ If pharmacological interventions can be administered early after irradiation, it may be possible to partially block or reverse the pathological changes in the alveolar epithelium.^25^ To date, no effective therapeutic drugs are available for RILI. Therefore, the development of efficient medications for the avoidance and treatment of RILI is urgently required. In this research, we constructed a mice model of RILI to explore the effect of DHA.

DHA is widely recognized as an effective drug against malaria. However, recent studies have shown that DHA may alleviate lung damage by reducing lung inflammation and fibrosis.^22^, ^26^ The precise mode of action of DHA in RILI is yet unclear, though. In a mice model of RILI, we observed structural damage and hemorrhage in the lungs and disruption of mitochondrial structures using H&E staining and TEM. Immunohistochemistry and ELISA experiments were used to detect the expressions of TNF‐α and TGF‐β. These pathological changes were ameliorated by the DHA treatment. The results showed that it was effective to ameliorate the pathological changes and attenuate the expression of TGF‐β and TNF‐α in the lung tissues with DHA treatment, which is in agreement with our prior experimental findings.^21^

Radiation therapy can limit GSH synthesis by generating large amounts of reactive oxygen species that deplete GSH and inhibit cysteine uptake, ultimately reducing GSH levels.^4^ And the reduction of GSH level triggers ferroptosis, which is consistent with our experimental results. Various studies have demonstrated that radiation therapy causes ferroptosis by suppressing GPX4, which in turn induces the development of RILI.^13^, ^27^, ^28^ A crucial regulatory enzyme in ferroptosis, GPX4's expression level frequently indicates the severity of ferroptosis.^5^ These results suggested that DHA considerably reduces radiation‐induced ferroptosis and effectively suppresses radiation‐induced pulmonary pathogenic alterations. Thus, ferroptosis might be crucial to RILI formation. These findings are in line with the findings of our study.

Our experiments demonstrated that the level of Nrf2 was mildly elevated in mice after radiation treatment, and the level of HO‐1 was elevated but not significantly different. This indicated that under stress conditions, Nrf2 and HO‐1 are compensating elevated to protect cells and tissues. The Nrf2 and HO‐1 levels in mice considerably increased after DHA administration. This indicated that DHA activates the Nrf2/HO‐1 pathway. In Sun et al.'s research on hepatocellular cancer, DHA increased free Nrf2 and enhanced the activation of the downstream target gene HO‐1.^29^ In the absence of stress, Nrf2 combines with Keap1 in the cytoplasm and is maintained at low levels.^30^ When subjected to external stimuli, Nrf2 separates from Keap1 and moves into the nucleus, activating various downstream genes, including HO‐1, as well as increasing the expression of GSH and acting as an antioxidant stressor.^31^, ^32^, ^33^ These results are in accordance with those of our study.

Our experimental results also revealed that both the protective effect of DHA on lung inflammation and the inhibition of ferroptosis were suppressed when Nrf2 inhibitors were used. We therefore speculated that the ability of DHA to inhibit cellular ferroptosis may be related to the inhibition of Nrf2 degradation. Several studies have demonstrated that many therapeutic agents suppress ferroptosis by triggering the Nrf2/HO‐1 pathway. In Li et al.'s study, in mice with acute pulmonary injury, panaxydol reduced ferroptosis via the Keap1‐Nrf2‐HO‐1 pathway.^33^ By triggering the Nrf2/HO‐1 signaling pathway, melatonin prevented ferroptosis caused by high glucose levels.^34^ Astaxanthin increases autophagy via activating the Nrf2/HO‐1 pathway and reduces liver injury by inhibiting ferroptosis.^35^ Maresin1 reduced ROS and activated Nrf2/HO‐1/GPX4 to prevent liver damage brought on by ferroptosis.^36^ We demonstrated in this study that DHA suppressed ferroptosis and decreased RILI by modulating the Nrf2/HO‐1 pathway. Several studies have shown that DHA can alleviate lung injury.^20^, ^22^ Some studies have demonstrated that DHA also has a mitigating effect on RILI,^37^ which is consistent with our findings.

However, there were certain restrictions on this study. Some studies have found that artemisinin has some toxicity,^38^ so is DHA, a derivative of artemisinin, to be toxic as well? DHA has been reported to treat kidney disease,^39^ but at the same time this author suggests that it is not clear whether it has other toxicities if used in long‐term, low‐dose therapy. These need to be explored in more studies to confirm the safety of DHA. In addition, our study was conducted only in vivo and was not validated in vitro. Our research offered preliminary evidence of the Nrf2/HO‐1 pathway's connection to ferroptosis, but it did not fully explore the mechanism of action. In addition to ferroptosis and the Nrf2/HO‐1 pathway's function in RILI control, there may be more regulatory mechanisms involved in the formation of RILI.

## 5 CONCLUSIONS

In this study, we found that post‐radiation mice showed significant lung injury and ferroptosis. Through the Nrf2/HO‐1 pathway, DHA prevented ferroptosis and reduced RILI. According to our research, ferroptosis and the Nrf2/HO‐1 pathway could be potential targets for the treatment of RILI, and DHA may be an applicable drug in RILI prevention and treatment.

## AUTHOR CONTRIBUTIONS

**Xin Ning**: Conceptualization; data curation; investigation; methodology; writing—original draft. **Weidong Zhao**: Data curation; methodology. **Qiaoyuan Wu**: Data curation; writing—review and editing. **Cailan Wang**: Data curation; methodology. **Shixiong Liang**: Conceptualization; project administration; supervision; writing—review and editing.

## CONFLICT OF INTEREST STATEMENT

The authors declare no conflict of interest.

## ETHICS STATEMENT

The animal procedures used in this study followed the standards for the Ethical Review of Animal Welfare in China (GB/T 35892‐2018) and the research obtained approval from the Animal Experimentation Ethics Committee of Guangxi Medical University (No. 202205013).

## Data Availability

Data generated or analyzed in this study are available from the authors upon reasonable request.
